# Global variation in sequencing impedes SARS-CoV-2 surveillance

**DOI:** 10.1371/journal.pgen.1009620

**Published:** 2021-07-15

**Authors:** Dana C. Crawford, Scott M. Williams

**Affiliations:** Departments of Population and Quantitative Health Sciences and Genetics and Genome Sciences, Cleveland Institute for Computational Biology, Case Western Reserve University, Cleveland, Ohio, United States of America; HudsonAlpha Institute for Biotechnology, UNITED STATES

## Viewpoint

Surveillance is essential to successful, rapid response of infectious disease outbreaks. While public health surveillance has historically focused on monitoring clinical cases and consequences of infection (e.g., case reports and hospitalizations) [1], technological advances in genomic sequencing rooted in the Human Genome Project and other large-scale investments in human genetics and genomic research and technologies now allow the unprecedented opportunity for pathogen surveillance down to base pair patterns of variation. Despite the availability and ubiquity of sequencing in several countries, the adoption of genomics as a strategy for pathogen surveillance has been slow, difficult, and inconsistent.

An extreme example of the slow adoption is that, as of early April 2021, the United States ranked 33th in the world in Severe Acute Respiratory Syndrome Coronavirus 2 (SARS-CoV-2) sequencing for variant surveillance, up from 36th a few weeks earlier [2–4]. For a country that led the sequencing of the human genome more than 2 decades ago, the lack of accrual of sequencing data in the midst of this pandemic is unbelievable and problematic. Capacity and expertise exist across several key academic centers and industry partners, yet these resources remained relatively dormant until recently with fewer than 7,000 new sequences the week of March 6 from more than 415,000 new cases in the same period [5–7]. At that point in time, several commentaries and interviews [2,3,8] provided factors associated with this genomic bottleneck, e.g., lack of funding, lack of robust sample tracking and data pipelines, and strict regulations governing biospecimen and data sharing. These variables, which are not unique to the US, have been successfully addressed by other countries such as Denmark and the United Kingdom (UK) [9]. Given the role of the US in developing and utilizing sequencing technologies in the study of human diseases, initial failure to extend this into the study of SARS-CoV-2 to track viral mutations and improve public health represents a troubling, self-inflicted barrier to battling Coronavirus Disease 2019 (COVID-19), especially in light of the high prevalence of SARS-Cov-2 infection in the US over the past year.

Historically, genomic studies in the US have been financially and scientifically well supported by several agencies including the US Department of Energy, National Institutes of Health (NIH), and private industry. Today’s mega-biobanks with electronic health records (EHRs) and other health-related data linked to DNA samples collected for genome-wide genotyping and whole genome sequencing are efforts supported by the Veterans Administration [10], medical centers, NIH, and private industry. Nearly absent from the US genomic-centric efforts is explicit collaboration with and investment by the primary public health agency, the Centers for Disease Control and Prevention (CDC). Maintaining zip code is more relevant to health than genetic code [11], the CDC did not initially prioritize genomic research with respect to public health. With the real time evolution of SARS-CoV-2 and the resulting impact on disease transmission and disease severity, this bias has created a gaping hole in our understanding of the trajectory of COVID-19. Recently, this has been at least partially addressed by new initiatives and funding through programs such as National SARS-CoV-2 Strain Surveillance (NS3) system. Initiated in November 2020, this program is partnering the CDC as of early 2021 with state health departments to process and sequence 750 samples per week and with commercial diagnostic labs to sequence 6,000 samples per week [12]. CDC is also working with 7 universities to conduct genomic surveillance research. In addition, the CDC-led SARS-CoV-2 Sequencing for Public Health Emergency Response, Epidemiology, and Surveillance (SPHERES) consortium has been developed to coordinate among more than 160 institutions active in SARS CoV-2 sequencing.

In stark contrast to the relatively uncoordinated and antiquated approach to SARS-CoV-2 genomic surveillance in America, successful public health sequencing surveillance programs outside the US embraced genomic technology early in the pandemic. China initiated this early by producing the first SARS-CoV-2 sequence [13], whose public widespread dissemination enabled near real-time worldwide sequence comparisons and the unprecedented rapid development of successful vaccines. The UK, ranked ninth in SARS-CoV-2 sequencing as of late January 2021, formed the COVID-19 Genomics UK (COG-UK) Consortium in April 2020 and have since sequenced more than 200,000 viral genomes, representing approximately 6% of reported COVID-19 cases in the UK [2,14]. As of April 2021, the UK climbed to fifth ranking with more than 8% of cases having been sequenced [4]. The COG-UK Consortium, financially supported by the Department of Health and Social Care, UK Research and Innovation, Wellcome, and Wellcome Sanger Institute, and the Consortium, includes several public health groups, universities, and others as their scientific partners [14]. Many of the same groups are core supporters of the UK Biobank, a cohort of 500,000 participants with genome-wide and health data available as a major worldwide research resource for health outcomes of interest, now including COVID-19 [15]. Meanwhile, the US had sequenced less than 0.5% of confirmed cases [2] with plans to ramp up sequencing [6,16], but this effort has coalesced only a year after the first US case of COVID-19 was confirmed in Washington State [17].

Although finances and limited supplies represented key impediments early in the US, this is not the case at present [4]. The CDC has recently committed more than $200 million to enhance the sequencing. Rather now the major issue may be that the US does not have an organized, ongoing population-based research cohort that can be leveraged for COVID-19 studies, genetic or otherwise, forcing investigators to scramble to form ad hoc consortia for the collection of data from electronic health records [18] or to augment existing public [19] and private [20] genomic collections with COVID-19 data. Data access is siloed and samples are held (or discarded [4]) by a plethora of disconnected labs, both public and private. This balkanization of the public health and testing efforts has not only slowed the process; it has substantially increased expenses. The White House recently announced a $1 billion dollar influx to increase sequencing capacity [21]. In comparison, the UK has had 2 major influxes of money into SARS-CoV-2 sequencing efforts totaling 20 million pounds in March 2020 producing more than 200,000 sequences [22] and an additional 12 million to produce sequence data from at least 20,000 cases per week. The results are clear; as of March 2021, the UK has generated approximately 40% of the SARS-CoV-2 sequences toward the global surveillance effort [23] for a fraction of the investment expected in the US.

Direct comparisons between non-US successful SARS-CoV-2 sequencing surveillance efforts and the US efforts are difficult and somewhat unfair given that the federal response to the pandemic was initiated under an administration that has since been replaced. Also, the American healthcare system and associated governmental agencies are mostly patchwork and disparate. The CDC, part of the US Department of Health and Human Services (HHS), typically leads disease surveillance and works in conjunction with other HHS agencies, such as the Indian Health Services, as well as public health agencies organized at the state level. The latter rely primarily on healthcare organizations for data on reportable diseases. Financial and technical resources at the state and local level can vary substantially, explaining in part why Washington State has sequenced 4.84% of their confirmed cases compared with just 0.45% in Ohio. The difference in sequencing observed between Washington State and practically every other US state may also be due to both the history of SARS-CoV-2 in the US and the existing public health genomics research activities [17,24–27].

Given that SARS-CoV-2 is a novel zoonotic disease with no prior human infections, sequencing and analysis inform both the trajectory of the outbreak as well as its evolution [28–30]. The opening of a new niche for the evolution of the virus makes tracking human borne mutations critical to our surveillance and control, as many of these mutations may not have been beneficial to the virus in other hosts and hence would not have survived earlier. This is of particular importance in areas with high incidence. For example, even though as of this writing, the rate of infection is waning in the US, due in part to vaccinations, it is raging in other parts of the world, such as India, with little to no access to vaccines. The current crisis in India and the past year’s tragedy in America has created two of among the largest viral populations in the world that can mutate into more transmissible [31] and more severe [32,33] versions of the original virus [34]. The emergence of B.1.1.7, B.1.351 [35], P.1 [36], among others, is a reminder that investments in SARS-CoV-2 genomics need to continue and be expanded as other variants are probably not be far behind given the worldwide variability in vaccination rates and adherence to COVID-19 precautions. Even though the US has set into motion funding and efforts to correct for its initial dearth of sequencing, the pipeline both in the US and globally will require additional and sustained support as the pandemic moves from locale to locale.

Apart from increased capacity for sequencing and analysis, provisions are also sorely needed to link genetic data to clinical and epidemiological data sources for public health research. These critical data linkages remain problematic in the US and resource-limited countries, but they are essential [37,38]. For countries with the adequate resources, increased sequencing capacity and the development of informatics and bioinformatics pipelines and workflows need to be adapted and adopted via international efforts. The pandemic is an evolving phenomenon, requiring worldwide genomic expertise and technology as part of effective SARS-CoV-2 surveillance. When linked to clinical and epidemiological data, the same expertise will help in understanding the factors relevant in variable host susceptibility and response to infection pre- or postvaccination, independent of and interacting with the genetic code of the evolving virus that knows no zip code or international boundaries.
